# Molecular dynamics simulations of methane adsorption and displacement from graphenylene shale reservoir nanochannels

**DOI:** 10.1038/s41598-023-41681-6

**Published:** 2023-09-22

**Authors:** Maryam Hajianzadeh, Jafar Mahmoudi, Sadegh Sadeghzadeh

**Affiliations:** 1 School of Chemistry, Damghan University, Damghan, Iran; 2 Nanotechnology Department, School of Advanced Technologies, Smart Micro/Nano Electro-Mechanical Systems Lab (SMNEMS), Iran University of Science and Technology, Tehran, Iran

**Keywords:** Energy science and technology, Engineering, Nanoscience and technology

## Abstract

Methane is the main component of shale gas and is adsorbed in shale pores. Methane adsorption not only affects the estimation of shale gas reserves but also reduces extraction efficiency. Therefore, investigating the behavior of methane adsorption in shale reservoirs is important for evaluating shale gas resources, as well as understanding its desorption and displacement from the nanochannels of shale gas reservoirs. In this research, molecular dynamics simulations were used to investigate the adsorption behavior of methane gas in organic shale pores made of graphenylene, followed by its displacement by CO2 and N2 injection gases. The effects of pore size, pressure, and temperature on adsorption were examined. It was observed that increasing the pore size at a constant pressure led to a decrease in the density of adsorbed methane molecules near the pore surface, while a stable free phase with constant density formed in the central region of the nanopore. Moreover, adsorption increased with increasing pressure, and at pressures ranging from 0 to 3 MPa, 15 and 20 Å pores exhibited lower methane adsorption compared to other pores. The amount of adsorption decreased with increasing temperature, and the observed adsorption isotherm followed the Langmuir adsorption isotherm. The mechanism of methane displacement by the two injected gases differed. Carbon dioxide filled both vacant adsorption sites and directly replaced the adsorbed methane. On the other hand, nitrogen only adsorbed onto the vacant sites and, by reducing the partial pressure of methane, facilitated the displacement of methane.

## Introduction

Currently, due to the growth of the world population and the advancement of technology, the need for energy has increased greatly and will continue to increase in the future^1^. Recently, natural gas has emerged as a major global energy source. Shale gas, primarily composed of methane, has become one of the most valuable unconventional natural gas sources due to its wide distribution, abundant reserves, and low pollution^2^. Shale gas is natural gas trapped in shale rock^3^. The main characteristic of shale gas reservoirs is very low permeability and porosity^3^. To extract the gas, increasing the permeability of reservoirs is a basic requirement. Hydraulic fracturing is the common method used to increase shale reservoir permeability, enabling the economic production of shale gas^4–6^. The United States of America was the first country to apply horizontal drilling and hydraulic fracturing in shale gas exploration and development^7^. Shale components consist of organic materials and inorganic minerals such as quartz, calcite, clay, etc^8^. Organic shale materials are mainly composed of a complex polymer called kerogen, which has nanometer-sized pores^9^. Kerogen primarily consists of carbon, hydrogen, nitrogen, oxygen, and small amounts of sulfur. The carbon content of kerogen ranges from approximately 70 to 90%^10^. Kerogen provides 50–60% of all gas adsorption sites and serves as the main source of shale gas^11,12^. Organic matter is a crucial factor in gas adsorption in shale matrix^7^. Organic porous media store more gas than inorganic porous media^13^. Methane adsorption not only affects the estimation of shale gas reserves but also reduces extraction efficiency. Therefore, research on methane gas adsorption on shale surfaces and its displacement using injection gases is of great importance^14^. The use of injection gases has become an efficient method for exploiting shale gas^15^. Carbon dioxide and nitrogen are commonly considered ideal gases for methane displacement^15^. However, global warming primarily results from the emission of carbon dioxide, which is a significant concern^7^. Some researchers have proposed the injection of carbon dioxide gas to displace adsorbed methane, offering several benefits such as improving shale gas recovery and simultaneously sequestering carbon dioxide in shale^16–19^. Compared to other techniques, using carbon dioxide for methane displacement is a practical, efficient, economical, and sustainable method for exploiting shale gas^20,21^.

Numerous studies have been conducted on methane absorption and its displacement in shale reservoirs using injection gases. Mosher et al. investigated methane adsorption isotherms in shale with different organic pore sizes. They reported that larger pores have a lower excess adsorption density than smaller pores. Smaller pore sizes reach their maximum excess adsorption density at lower pressures, while larger pores exhibit a broad, indistinct peak at higher pressures^22^. Chen et al. investigated methane adsorption on the heterogeneous surface of shale, which combined organic and inorganic components. They found that the graphene surface exhibited a much stronger adsorption capacity than montmorillonite due to higher gas density near the graphene surface. This phenomenon indicates a significant difference in the interaction between gas-organic matter and gas-inorganic matter^23^. Zhang et al. used Monte Carlo simulations and molecular dynamics to study the surface adsorption properties of methane and ethane in the inorganic pores of illite. They reported that the adsorption of methane and ethane increased with increasing pressure, and their adsorption isotherm followed the Langmuir adsorption isotherm^24^. Nan et al. studied the adsorption of methane molecules and the sliding length in organic graphite nanochannels under shale reservoir conditions using Monte Carlo simulations and molecular dynamics. They reported that at large pore sizes, the density in the middle of the pore converges to the bulk, while such convergence is not observed in small pores, and only adsorption layers are present^14^. Liu et al. investigated the adsorption and emission of a binary mixture of carbon dioxide and methane in an organic graphene nanochannel. They reported that under the same conditions, carbon dioxide has a stronger adsorption capacity than methane on graphene nanochannel surfaces. This finding indicates that the addition of carbon dioxide can remove adsorbed methane from the graphene nanochannel surfaces^25^. Wang et al. investigated the average isosteric heat of the binary mixture of carbon dioxide and methane in kerogen shale nanoparticles and found that the isosteric heat of carbon dioxide is higher than that of methane at the same temperature and pressure. This indicates that the kerogen models favor carbon dioxide over methane^26^. Yuan et al. investigated the molecular dynamics simulation for the displacement of methane molecules by injecting carbon dioxide molecules on the graphene surface. They found that when carbon dioxide approaches the graphene surface vertically, the displacement of methane occurs easily^14^. Shi et al. studied the displacement of methane with carbon dioxide and nitrogen injection gases and observed that both types of gas led to a drastic reduction in methane loading, with carbon dioxide displacing methane in the adsorbed layer more effectively than nitrogen^14^. Using molecular dynamics and Monte Carlo simulations, Sun et al. investigated the displacement of adsorbed methane by carbon dioxide in kerogen nanopores. They reported that as the bulk carbon dioxide pressure increases, more methane is displaced, and carbon dioxide is adsorbed and stored in the kerogen nanopore during the displacement process^27^.

In this research, the mechanism of methane adsorption and displacement in carbon pores has been investigated through molecular dynamics simulation. To study shale, it is necessary to simplify the complex structure of the shale matrix to gain fundamental insights into its surface adsorption behavior^7^. Since organic porous media store more gas than inorganic porous media^13^, organic pores have been used to investigate methane adsorption. Kerogen, with its carbon skeleton, is generally considered hydrophobic and has been simulated using carbon materials such as graphene and graphite for simplicity^28^. Polycyclic aromatic hydrocarbons are believed to be the main component of shale matrix organic matter, especially in shale gas reservoirs^7^. Graphenylene, a two-dimensional carbon allotrope with a unit cell consisting of two six-membered carbon rings connected by a four-membered ring, closely resembles the structure of kerogen. The use of heterogeneous surfaces like graphenylene sheets is a modeling technique employed to simplify and approximate the intricate structure of shale pore walls. Shale formations are indeed highly complex and consist of various components, However, when studying shale formations, it is often challenging to directly simulate the full complexity of these pore structures due to computational limitations. According to the available sources, graphenylene has not been previously used for simulation to investigate methane adsorption behavior in shale reservoirs. Therefore, in this research, graphenylene is used to investigate the adsorption behavior of shale gas.

Shale gas mainly consists of methane (70–90%) along with other light hydrocarbons such as ethane, propane, and butane (0–20%), as well as water, carbon dioxide, nitrogen, and trace amounts of hydrogen sulfide (0–5%)^29^. Since methane is the primary component of shale gas, it is used to investigate the behavior of shale gas^7^. Considering that there are a large number of nanoparticles in the organic materials of shale reservoirs where the pore diameter is less than 10 nm, and they serve as the main storage space for shale gas adsorption^30^, the simulation for the pore size of H = 7,10,15 and 20 Å is done. Additionally, the effect of a wide range of pressures (1, 2, 3, 4, 6, 8, 10, 12, 16, and 20 MPa) and temperatures (298, 320, and 350 K) on the adsorption isotherm and isosteric heat of methane adsorption was investigated. This comprehensive investigation allows us to gain insights into the adsorption and desorption mechanisms at different pore sizes, which is crucial for understanding the overall behavior of methane in shale formations. Finally, carbon dioxide and nitrogen injection gases were used to displace the adsorbed methane, and the mechanisms of methane movement by these two gases were compared.

## Model and simulation method

Molecular dynamics simulations using the LAMMPS software^31^ were employed to investigate the adsorption and movement of methane in graphenylene nanopores.

### Initial configuration of the system

**Figure 1:**
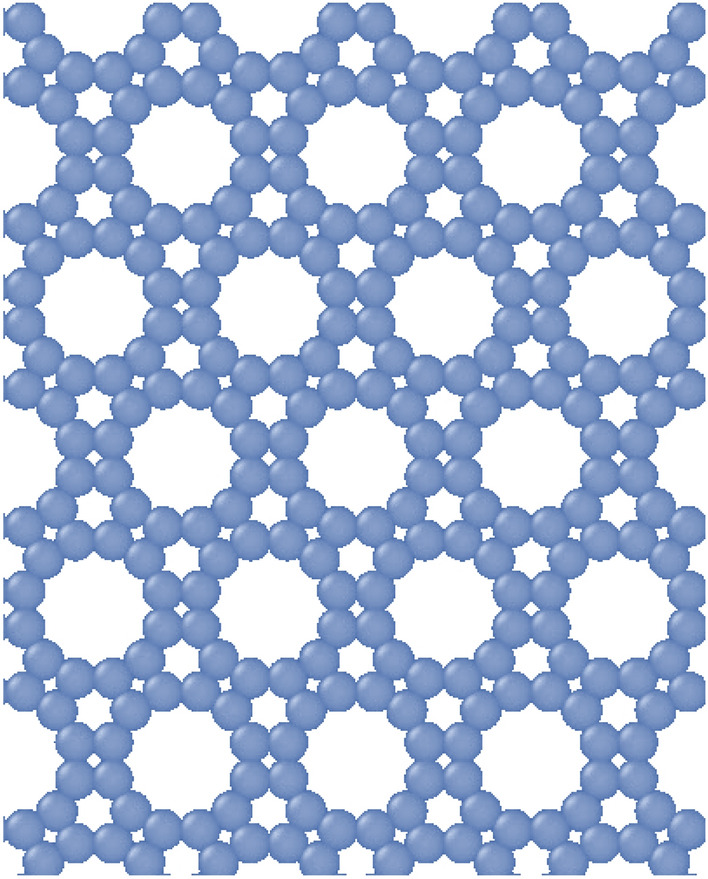
Graphenylene structure created.

The first step in conducting the simulation is to model the simulation box, including the coordinates of atoms and molecules, which consist of graphenylene sheets and methane molecules. To create the graphenylene sheet, the coordinates of its unit cell must be obtained, and then it is expanded to the desired dimensions for simulation in all directions. For this purpose, the atomic coordinates of the graphenylene unit cell were obtained as shown in Fig. 1.

To perform a molecular dynamics simulation, an initial configuration must be created, which needs to be carefully selected. In this simulation, the number of methane molecules corresponding to different pressures was obtained using the Van der Waals equation of state. Packmol software was used to create the initial configuration and data file for methane in the molecular simulation. During the adsorption stage, there is only one methane molecule in the system, and the existing bond is solely the C-H bond of methane. However, during displacement and desorption, carbon dioxide or nitrogen are also present in addition to methane, and C = O and N≡N bonds are added. Packmol software is not responsible for creating the data file at this stage because it does not differentiate between the C-H bond in methane and the C = O bond in carbon dioxide. For this reason, during the moving stage, VMD software is used to create a data file that includes both the bond angle and type.

**Figure 2:**
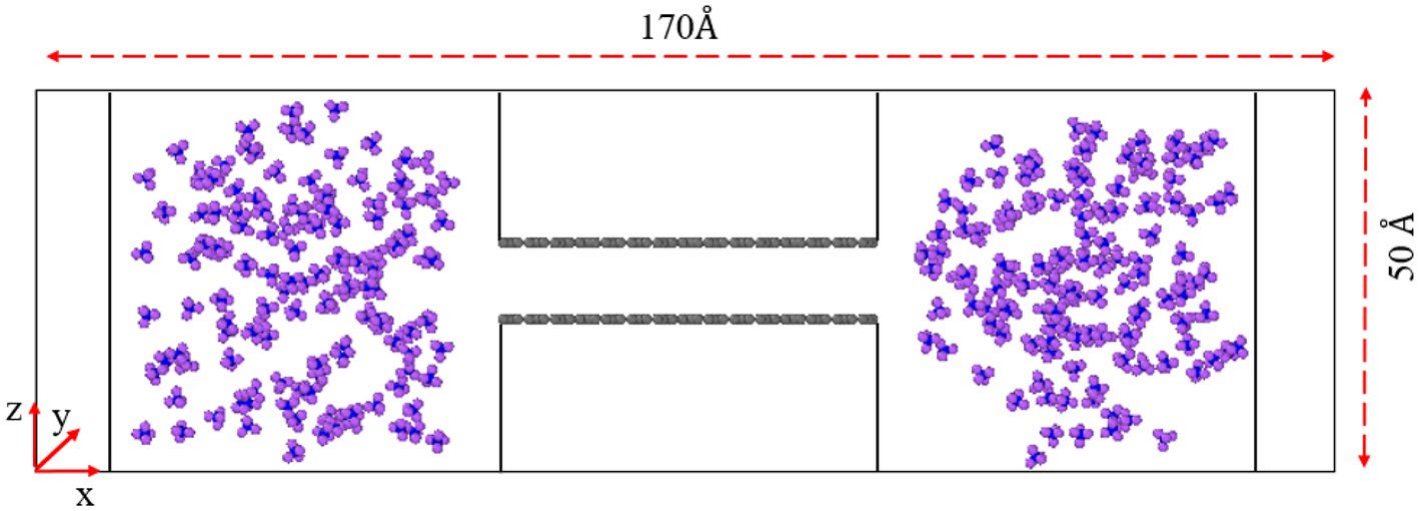
Overview of the simulation box: Methane molecules are placed in the tanks, with methane hydrogen shown in purple, methane carbon in blue, and graphenylene carbon in gray.

Two graphenylene sheets, formed by carbon atoms independently and parallel to each other, are modeled as organic shale gap pores and remain fixed during the simulation. The distance between the two graphenylene sheets (H) is set to model different pore sizes. The simulation box used in this research is divided into three equal parts and has dimensions of 50 Å × 50 Å × 170 Å. The left and right tanks, along with the simulated channel connecting them, form the structure. The simulation box is depicted in Fig. 2. The left and right walls of the tanks are considered mirror walls (reflective) so that molecules colliding with the walls are returned without interacting with the wall.

In this simulation, the number of methane molecules corresponding to different pressures was obtained using the Van der Waals equation, as given in Eq. (1):

1 $$\left(\mathrm{P}+\frac{{\mathrm{an}}^{2}}{{\mathrm{V}}^{2}}\right)\left(\mathrm{V}-\mathrm{nb}\right)=\mathrm{nRT}$$

The pressure is determined by the density of methane in the bulk phase (reservoir), where "a" and "b" are Van der Waals coefficients that have fixed and specific values for each gas. "R" is the general gas constant, "P" is the pressure, "V" is the volume, "n" is the number of moles, and "T" is the temperature of the gas. Methane molecules are placed in the left and right tanks, and due to the pressure in the tanks, the molecules enter the gap and have a tendency to be adsorbed on the walls.

To simulate the movement of methane adsorbed by injection gas, methane is first adsorbed in the hole, and carbon dioxide and nitrogen injection gases are placed in the left tank. Due to the pressure difference, the injection gases enter the pore and displace the adsorbed methane. In this research, the pressure of methane adsorbed in the pore was 3.12 MPa, and the injection gas pressure was considered as 3.588 MPa for both carbon dioxide and nitrogen based on the conditions that are commonly encountered in shale gas extraction operations. Boundary conditions were applied as periodic along the z, y, and x-axes.

In this simulation, the interaction between carbon and hydrogen atoms of methane is modeled using the Airebo potential, which is commonly used for hydrocarbons, with a cut-off radius of 3 Å. For the intermolecular interactions between graphenylene carbon atoms and carbon dioxide and nitrogen molecules, the 6–12 Lennard–Jones potential with a cut-off radius of 10 Å was used, as shown in Eq. (2):.

2 $${U}_{ij}=4{\varepsilon }_{ij} \left[{\left(\frac{{\sigma }_{ij}}{{r}_{ij}}\right)}^{12}- {\left(\frac{{\sigma }_{ij}}{{r}_{ij}}\right)}^{6}\right]$$

In this equation, "r_ij" is the distance between two atoms "i" and "j", "ε" is the interaction intensity parameter and the depth of the potential well, and "σ" is the molecular length scale where the intraparticle potential is zero. The values of parameters "ε" and "σ" used in this simulation are listed in Table 1.

**Table 1:** Required values of ε and σ for the Lennard–Jones potential^32–34^.

	$$\varepsilon$$(eV)	*σ* (Å)
C_(CH4)_	0.00474427	3.4
H_(CH4)_	0.00068085	2.65
C_Graphenylene_	0.00241104	3.4
C_(CO2)_	0.00242397	2.757
O_(CO2)_	0.0069375	3.033
N_2_	0.00313671	3.32

**Figure 3:**
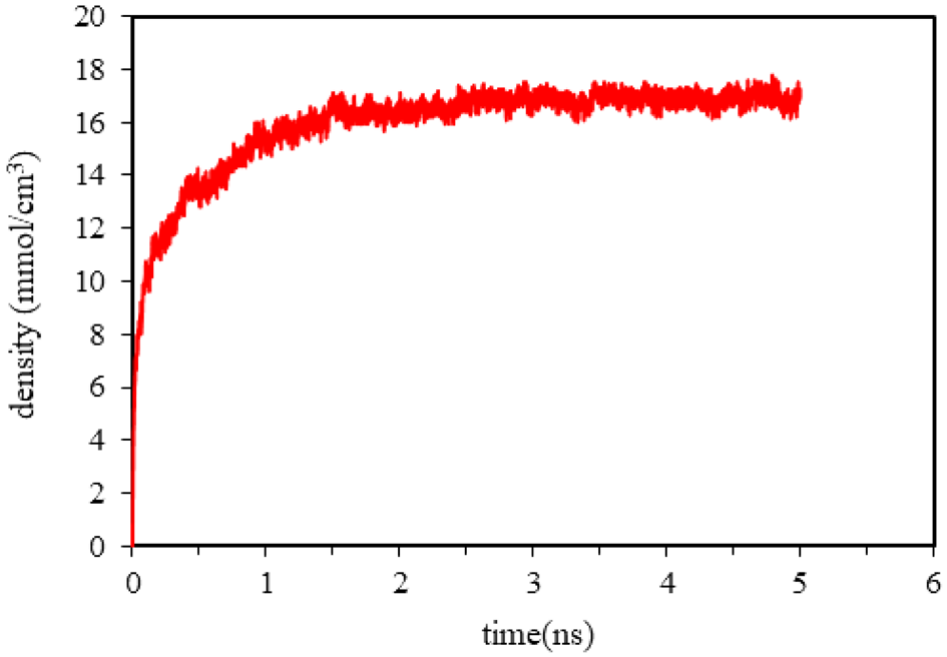
The density of methane absorbed in the Graphenylene pore over time.

The interaction between different types of atoms can be calculated using the Lorentz-Bertlet mixing law. All simulations are performed using the NVT algorithm with a time step of 0.5 femtoseconds. The temperature is controlled by a Noz-Hoor thermostat^25^. In each simulation, the first 2 ns were used for system equilibration (relaxation) and the next 0.5 ns were used for data collection. These steps include monitoring the potential energy, temperature, and pressure fluctuations of the system over time. By assessing these parameters, we can verify whether the system has reached a steady state before proceeding with the data collection step. From a computational point of view, equilibrium in the system is observed when the controlled thermodynamic quantities reach an acceptable stability. In this simulation, the density of methane molecules in the pore was controlled for five nanoseconds and it was observed that it takes 2 ns for the system to reach stability in density and equilibrium. These results are presented in Fig. 3. Based on the findings, it can be observed that the first 2 ns of the simulation is sufficient to balance the system.

## Results and discussion

### Methane adsorption in the pores

**Figure 4:**
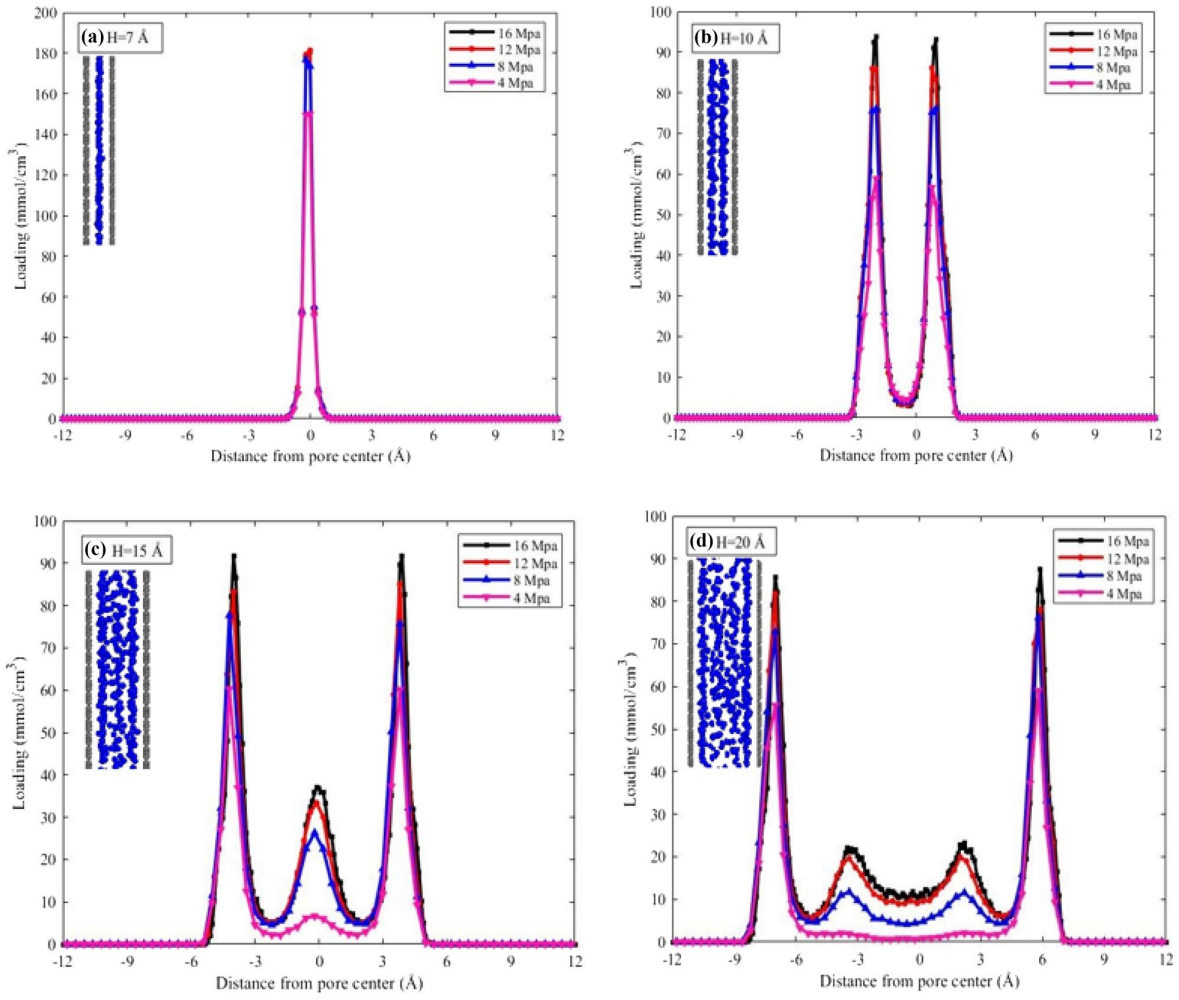
Density distribution and methane adsorption structures in different pores.

To investigate the effect of pore size on shale gas adsorption, the density distribution of methane molecules in pores of different cracks is shown in Fig. 4. Four pore sizes of 7, 10, 15, and 20 Å, and pressures of 4, 8, 12, and 16 MPa have been investigated. The results show that methane molecules tend to accumulate near the wall due to the strong interaction between the fluid and the surface, forming an adsorption layer. Additionally, the peak density is proportional to the pressure, and as the pressure increases, all density peaks also increase.

The density distribution for a pore size of 7 Å in Fig. 4a shows that methane molecules form a single adsorption layer in the center of the pore, which is related to the potential energy. In small pores, due to the close proximity of the walls, the force fields of the pore walls overlap and reinforce each other, resulting in the highest density distribution that significantly affects the adsorption behavior. However, the dynamic diameter of a methane molecule is 3.8 Å^35^, which prevents it from being closer to one of the walls in the 7 Å pore size, keeping it located in the center of the pore.

In the 10 Å pore size shown in Fig. 4b, methane molecules form two main adsorption layers on the surface of the pore wall due to the strong interaction between the pore walls and methane molecules. With an increase in pore size, the distance between the two walls increases, and the potential of the walls that were previously coinciding starts to separate, resulting in the appearance of two symmetrical peaks.

When the pore size increases to 15 Å, a much weaker peak gradually appears in the density distribution at the center of the two main adsorption layers, as shown in Fig. 4c. This indicates a lower gas accumulation compared to the first adsorption layer, and some molecules have entered the free phase due to saturation of the adsorption sites on the pore surface.

In the methane density distribution diagram for the 20 Å pore shown in Fig. 4d, two sub-peaks with low density are observed in addition to the main layers, which become more noticeable at high pressures. With an increase in pore size, more molecules enter the pore, and after the adsorption sites on the surface are saturated, molecules enter the free phase. In this case, both free phase gas molecules can be extracted and utilized. Moreover, due to the lack of potential overlap between the pore walls, the efficiency of the displacement process increases. Generally, increasing the pore size affects the arrangement of molecules, and they tend to access areas further from the surface^29^. The interaction between the pore and gas molecules weakens with an increase in pore size. It was observed that the amount of gas adsorbed on the surface has an inverse relationship with the diameter of the pores, and the lowest amount of adsorption on the pore surface is observed in the largest pore and at the lowest pressure. These findings are consistent with other articles^15,29^.

**Figure 5:**
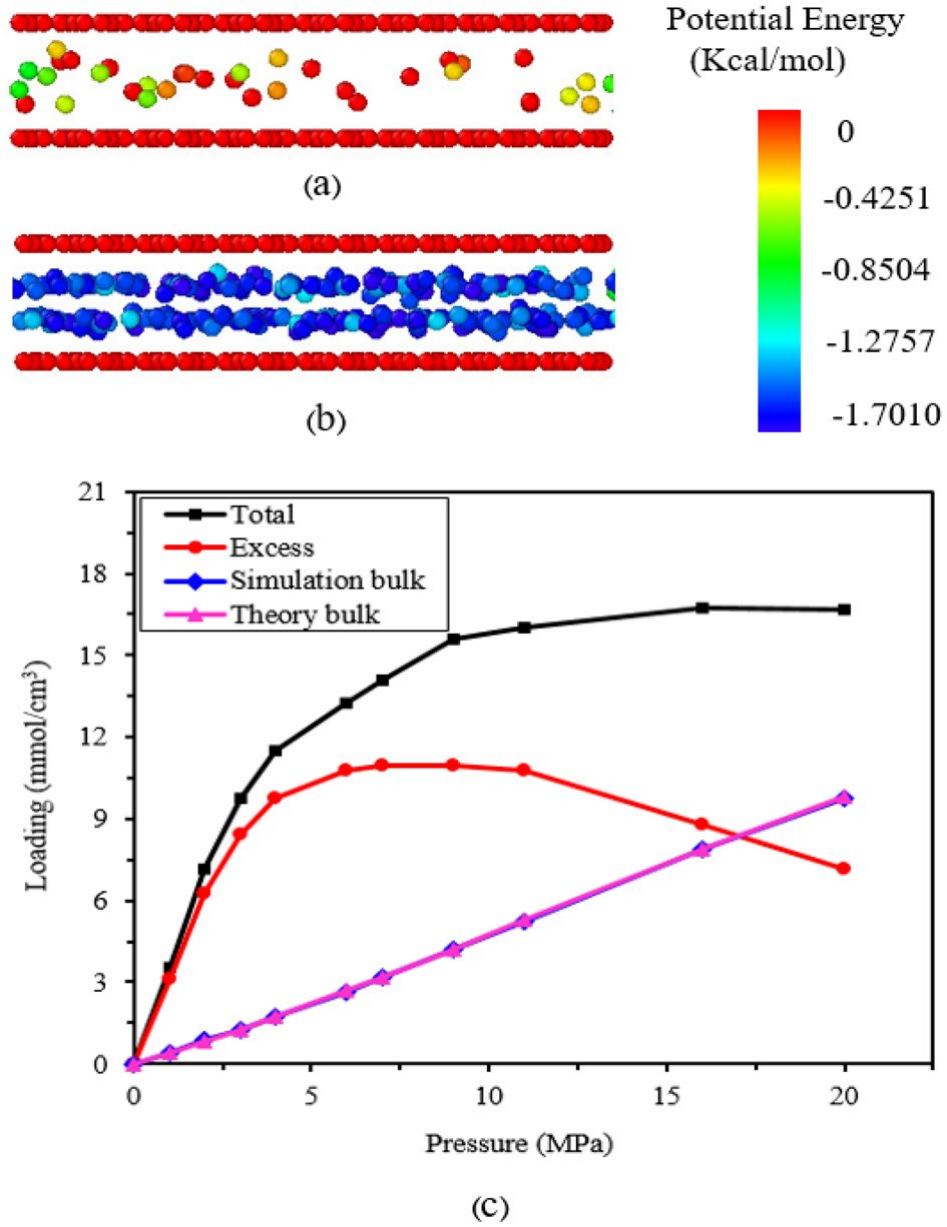
(a) Free phase, (**b**) adsorbed phase; Spheres represent carbons of methane with colors indicating different values of molecular potential energy. (**c**) Total, excess, and free adsorption of methane in a 10 Å pore at 298 K.

In this research, to investigate the effect of pressure, different pressures ranging from 1 to 20 MPa were considered for a pore size of 10 Å. The observed potentials between methane molecules and the pore wall contribute to methane adsorption. As Fig. 5a shows, when there is no interaction potential between the methane molecules and the pore wall, the methane molecules exist in the free phase within the gaps, and their potential is negligible, almost zero. When the interaction potential takes effect, as shown in Fig. 5b, more methane molecules enter the gap pores and adsorb onto the pore surface. In this case, the potential energy of the adsorbed methane decreases.

Total adsorption, free gas content, and excess adsorption are different terms used in the literature to describe adsorption. Total adsorption is defined as the total amount of gas present in the pore space at a given temperature and pressure. Free gas is not affected by the pore walls or any neighboring gas molecules. In other words, it refers to the gas in the pore when there is no potential function, and it represents a region where adsorbed molecules can move freely due to the negligible surface effect^14^. Excess adsorption is the difference between total gas adsorption and free gas content and can be obtained using the following equation:

3 $${N}_{ex}={N}_{tot}-\frac{{\rho }_{g}V}{M}$$

where N_ex is the amount of excess adsorption, N_tot is the amount of total adsorption, ρ_g is the density of the free gas, which is calculated using available data from the National Institute of Standards and Technology (NIST) website, V is the free volume of the pore, and M is the molecular weight of the adsorbed molecule^24^.

Figure 5c illustrates the isotherm of total adsorption, excess adsorption, and methane mass phase. At the same pressure, the total density is higher than the bulk (free) density due to adsorption. The free density of methane (bulk) is obtained from data collected on the National Institute of Standards and Technology (NIST) website, which corresponds to the free density of the methane molecule obtained from this simulation, thereby confirming the accuracy of the model and method. As the pressure increases, both total adsorption and excess density increase initially, then decrease with further increases in pressure. This process demonstrates that there is an optimal pressure for the maximum amount of adsorption, which is crucial for gas production. At low pressure, adsorption is minimal, but as the system pressure increases, the gas experiences strong adsorption forces from organic solids, leading to surface adsorption on the solid surface^13^. As the pressure continues to rise, the adsorption sites on the adsorbent surface become filled and saturated. The pressure increases until a certain point where the pressure required to add one more gas molecule to the adsorbed phase equals the pressure required to add one more molecule to the bulk phase. At this point, no further molecules are added to the adsorbed state, representing the maximum amount of excess adsorption. Beyond this point, increasing pressure without adding to the adsorbed layer results in an increase in bulk phase density. Consequently, the bulk gas density increases faster than the adsorbed gas density, leading to a decrease in additional gas adsorption. Since no molecules are added to the adsorbed layer, the relative difference in density decreases towards zero following the isotherm. These findings are consistent with those of other sources^13,22,24^. Furthermore, an increase in pressure enhances the interaction between gas molecules and the wall surface, resulting in increased adsorption behavior^23^.

The amount of gas adsorbed in shale rock is a function of pressure. With a decrease in pressure near production wells and fractures connected to the well, the adsorbed gas transitions into the free gas phase^30^, which can be extracted and utilized. Therefore, lower pressures are more favorable for reduced adsorption and increased desorption of methane molecules.

**Figure 6:**
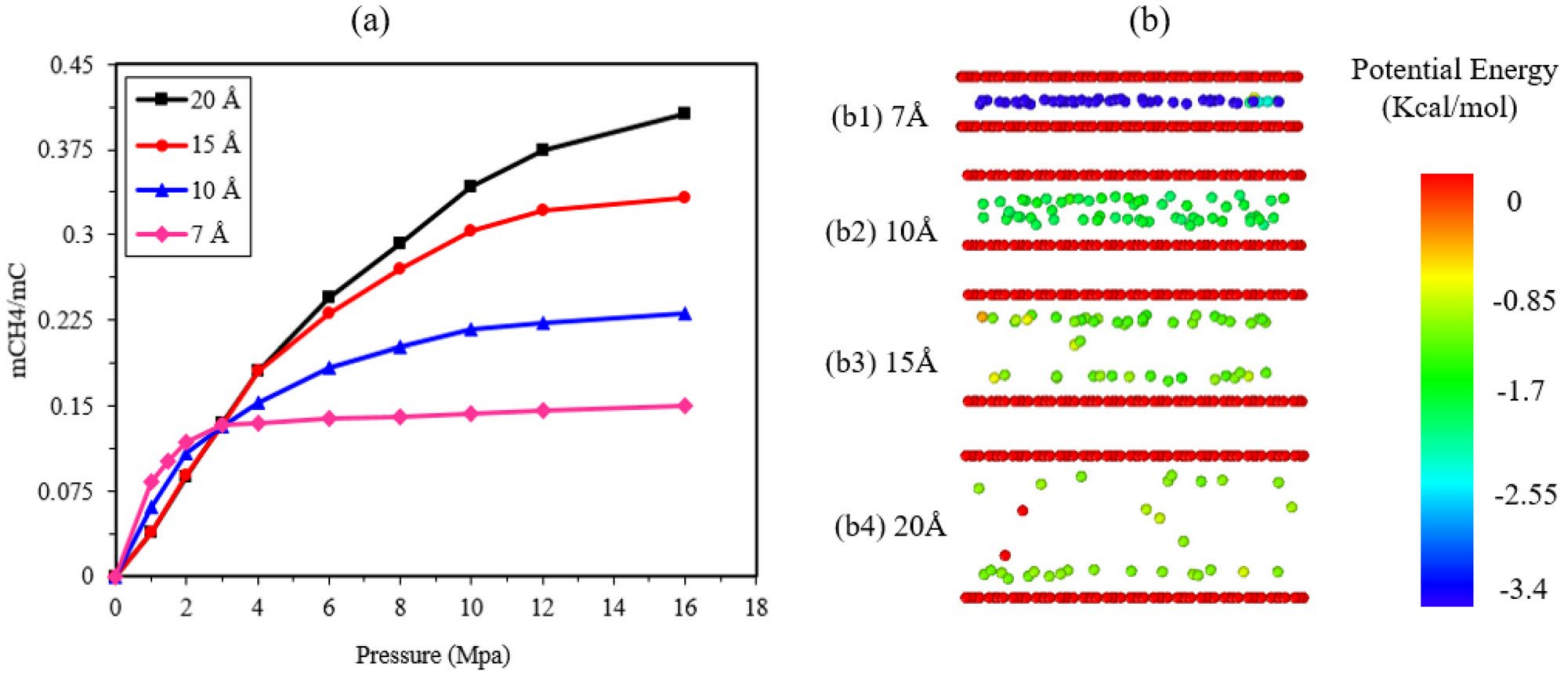
(**a**) Adsorption isotherm for four slit holes in K 298 (**b**) Distribution of potential energy in four slit holes with pressure equal to 1 MPa; Colors represent different values of molecular potential energy.

The adsorption isotherm is an important aspect of the adsorption process. In this research, the adsorption isotherm, which shows the amount of adsorbed methane against pressure for pore sizes of 7, 10, 15, and 20 Å at a temperature of 298 K, is depicted in Fig. 6a. The amount of adsorbed methane is normalized by the mass of adsorbed methane divided by the mass of pore carbons, represented by graphenylene. The simple Langmuir model is often used to describe the behavior of surface adsorption systems. Despite the heterogeneity of gas adsorption sites in shale, gas adsorption in shale can be adequately described by the Langmuir adsorption isotherm model^36^. In adsorption isotherms, the amount of substance adsorbed increases with increasing pressure until a monolayer is formed on the surface. Further increases in pressure result in the formation of more than one layer on the surface. Joe and his colleagues studied the interaction between methane and shale chemical compounds and plotted the radial distribution function (RDF). They found that the RDF lines exhibit only one adsorption peak, indicating monolayer adsorption. The existence of very irregular pores and limited transparency accounts for the absence of a second layer^37^. Furthermore, the adsorption of methane molecules on the shale pore surface is physical adsorption driven by van der Waals forces, aligning with the Langmuir adsorption isotherm that describes physical adsorption and single-layer adsorption of gas molecules on solid surfaces.

The results in Fig. 6a demonstrate that, at the same temperature, the amount of adsorbed methane continuously increases with increasing pressure in all pores. This is attributed to the growing interaction force between methane molecules and the pore walls as the pressure rises, leading to greater adsorption on the pore surfaces. Initially, this occurs at a faster rate, followed by a slower rate after the formation of the first adsorption layer. Increasing the pore size expands the gas space within the pores, resulting in a higher total adsorption. Small pores quickly reach saturation due to their narrow pore plane spacing, while large pores remain unsaturated even at relatively high pressures, allowing methane molecules to continue filling the free zone.

In the low-pressure region (0–3 MPa), small pores exhibit a higher initial velocity compared to large pores, and the amount of adsorption in the 7 Å pore size is greater than in other pores due to the overlapping force fields of the pore walls and the lowest potential energy, as shown in Fig. 6b. Pore sizes of 15 and 20 Å have the same amount of adsorbed methane in this pressure range because they possess the same potential energy. As mentioned earlier, in large pores, the greater distance between pore plates separates the potential of the pore walls into two mono-potential systems near each surface, resulting in less adsorption of methane molecules. At higher pressures, the amount of adsorption is directly related to the increase in pore size. In larger pores and at higher pressures, there is more space for methane molecules, leading to increased total adsorption and greater interaction between the gas and pore surfaces. By increasing the pore size, more molecules can enter the bulk area.

**Figure 7:**
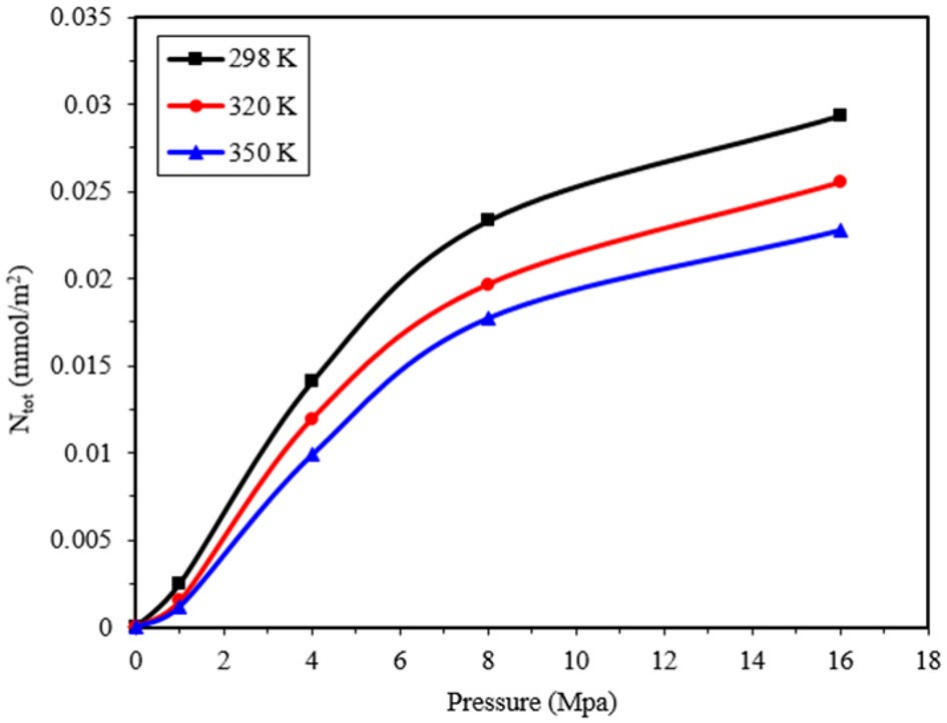
The graph illustrates the amount of adsorption of methane molecules at different temperatures: 298, 320, and 350 K.

There is a close relationship between the adsorption of gas molecules and temperature. Therefore, the effect of temperature has also been investigated in this research. Simulations were conducted at temperatures of 298, 320, and 350 K to examine the thermal behavior of gases. Pressures of 1, 4, 8, and 16 MPa were applied to the 20 Å pore, which had a lower density of methane molecules, as depicted in Fig. 7. As the temperature increased, the total adsorption of methane molecules decreased. The effect of temperature is more pronounced at higher pressures due to the larger number of methane molecules in the system. Kinetic energy is temperature-dependent, and higher temperatures result in greater kinetic energy and thermal motion of gas molecules. This leads to the escape of some adsorbed methane molecules from the pore surfaces toward the center of the nanopore, increasing the release of gas molecules. Liu and his colleagues also reported a decrease in the adsorption rate at high temperatures due to increased molecular kinetic energy. This increase in temperature corresponds to an increase in the molecular diffusion coefficient, which aligns with experimental findings^14^. The rise in temperature intensifies the thermal movement of molecules, increasing spatial hindrance and the probability of molecule collisions. Consequently, the duration of molecules staying on the graphenylene surface is reduced, resulting in reduced adsorption.

The adsorption of methane molecules in graphenylene pores follows the Langmuir adsorption isotherm. Equation (4) is utilized to fit the adsorption isotherm lines in the Langmuir model, which is the most common adsorption isotherm. The equation is as follows:

4 $$N= {N}_{max}\frac{bP}{1+bP}$$

**Figure 8:**
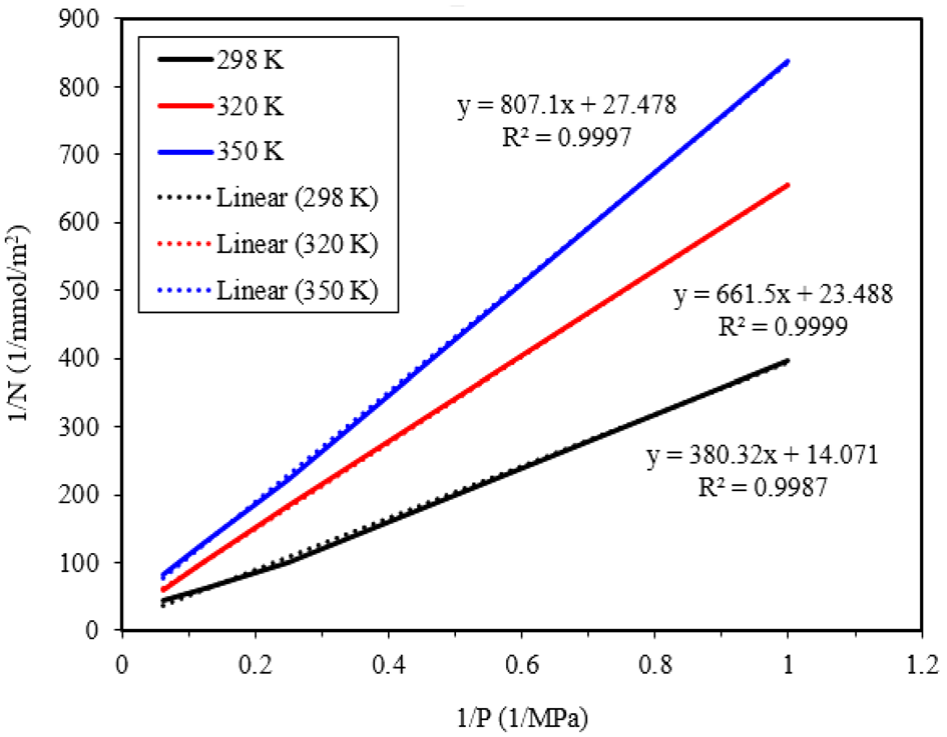
Adsorption fitted diagram for methane in the Langmuir adsorption isotherm equation.

Here, N represents the gas adsorption value, N_max is the maximum Langmuir surface adsorption value (LMAA), b is the Langmuir constant (1/MPa), and P is the pressure (MPa). To obtain N_max and b, a linearized version of Eq. (4) was employed, plotting 1/N against 1/P at different temperatures. The results are presented in Fig. 8 and Table 2. Based on the findings, it can be observed that both the maximum Langmuir surface adsorption value and the Langmuir constant decrease with increasing temperature.

**Table 2:** Adsorption fitted results for pure methane in the Langmuir adsorption isotherm model.

دما (K)	*N*_*max*_ (mmol/m^2^)	*b* (1/MPa)	Fitted linear equation	R square (*R*^*2*^)
298	0.07107	0.037	y = 380.32 x + 14.071	0.9987
320	0.04257	0.03551	y = 661.5 x + 23.488	0.9999
350	0.03639	0.03405	y = 807.1 x + 27.478	0.9997

To further investigate the differences in the adsorption capacity of methane molecules in different pore sizes, the isosteric heat (Qst) in Eq. (5) has been used to check the adsorption properties. The isosteric heat of adsorption evaluates the affinity between the adsorbent and the adsorbed substance, indicating the strength of the energetic interaction. The equation for Qst is as follows:

5 $${Q}_{st}= {R}_{g}T-\frac{\langle {N}_{ad}{U}_{ad}\rangle -\langle {N}_{ad}\rangle \langle {U}_{ad}\rangle }{\langle {N}_{ad}^{2}\rangle -{\langle {N}_{ad}\rangle }^{2}}$$

**Figure 9:**
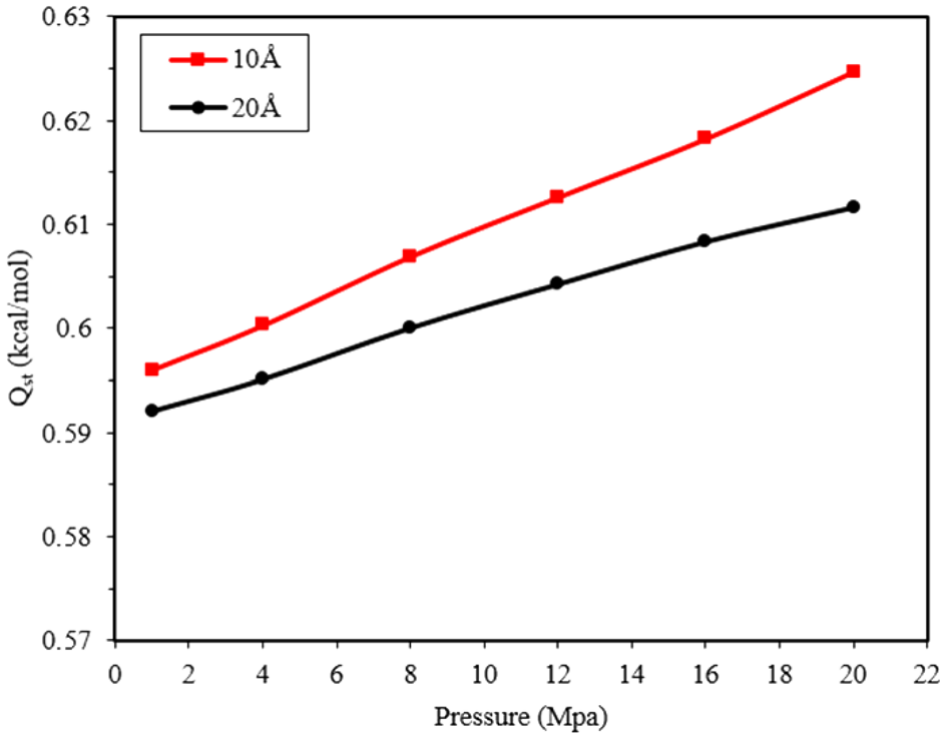
Isosteric heat of methane adsorption in 10 and 20 Å nanopores.

Here, <  > denotes the average of the set. Rg is the gas constant, T is the temperature, Nad is the number of adsorbed molecules, and Uad is the surface adsorption energy. The first term represents the contribution of molecular thermal energy, while the second term represents the interaction energy of the adsorbing-adsorbed substance. The isosteric heat of adsorption (Qst) for methane with pore sizes of 10 Å and 20 Å was calculated to compare the adsorption capacity of pores at pressures ranging from 1 to 20 MPa and a temperature of 298 K. The results are shown in Fig. 9. The findings indicate that the isosteric heat of adsorption (adsorbed-adsorbent interaction) in pure methane is influenced by pressure and different pore sizes. A higher isosteric heat of adsorption corresponds to a stronger interaction between the adsorbed material and the adsorbent. The isosteric heat of adsorption for pure methane decreases as the pore size increases from 10 Å to 20 Å. The 10 Å pore exhibits a stronger adsorption capacity and a more robust interaction between methane and the pore surface due to a higher isosteric heat. These results align with similar studies^24,38^, where Lee et al. also reported a continuous increase in the isosteric heat of adsorption with increasing adsorption capacity^39^. As pressure increases, the interaction between gas molecules and the pore wall intensifies, leading to an elevated methane adsorption rate. Consequently, the isosteric heat contribution of methane increases linearly within the pores. This implies that methane adsorption capacity continues to grow at high pressure.

Figure 9 shows that the slope of the isosteric heat curve corresponding to the 10 Å pore size is higher than the slope of the curve for the 20 Å pore size. This indicates stronger intermolecular interactions between the methane molecule and graphenylene carbon within the 10 Å pore.

### Displacement of adsorbed methane

**Figure 10:**
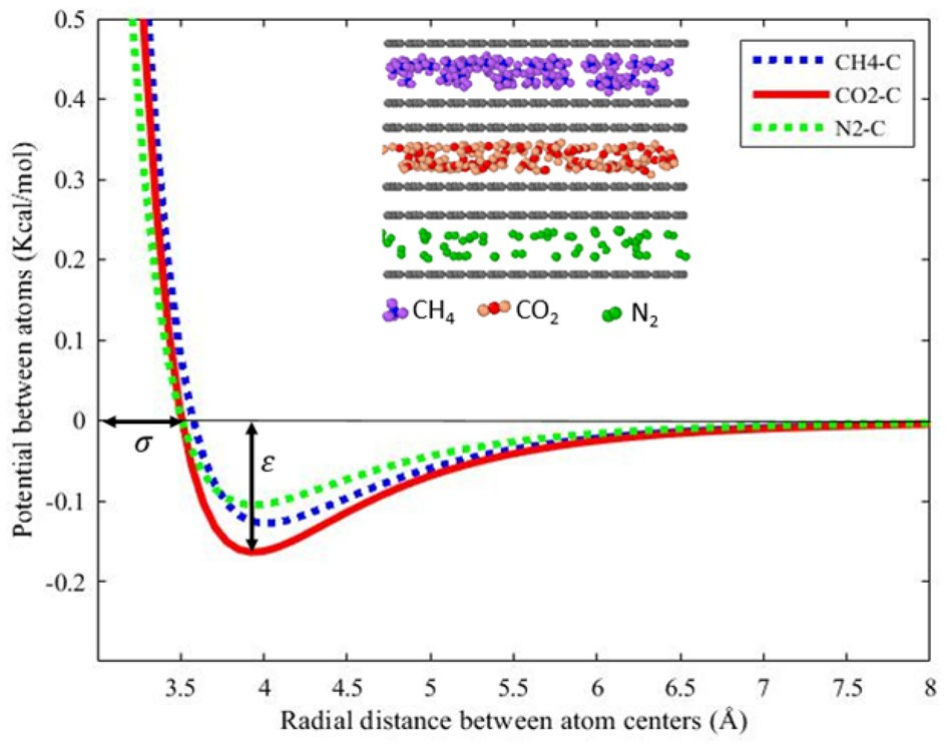
Lennard–Jones chart CH_4_-C, CO_2_-C, and N_2_-C at the same temperature and pressure.

Carbon dioxide and nitrogen injection gases are typically considered ideal gases for displacing methane^14^. In this section, we investigated the adsorption capacity of three gases: methane, carbon dioxide, and nitrogen. Results from various studies indicate that the adsorption capacity of gases is influenced by the adsorption potentials between the gases and shale matrix atoms. The adsorption potential between gases and carbon atoms is characterized by the Lennard–Jones potential, which is defined in Eq. (2) and depicted in Fig. 10. In this context, ε represents the minimum potential energy of interaction between two particles, signifying the depth of the potential well and indicating the strength of attraction between the particles. σ, on the other hand, represents the distance between particles where the potential energy becomes zero. ε serves as a measure of adsorption capacity, with higher values corresponding to greater gas adsorption capacity. Figure 10 illustrates that the potential energy of carbon dioxide is higher than that of methane, and the potential energy of methane is higher than that of nitrogen. Consequently, the adsorption capacity of the three gases can be ranked as CO2 > CH4 > N2. The values of ε for CO2, CH4, and N2 are 0.1639, 0.1278, and 0.1049 kcal/mol, respectively, as reported in Table 3. A study conducted by Yuan et al.^14^ provides an energy comparison between methane or carbon dioxide and the carbon surface of graphenylene. Higher adsorption capacity leads to a greater number of gas molecules being adsorbed in the pores. Carbon dioxide exhibits a higher adsorption capacity compared to methane, while nitrogen's adsorption capacity is lower than that of methane^14^. Therefore, the mechanisms of movement of methane adsorbed by carbon dioxide and nitrogen are compared.

**Table 3:** Lennard–Jones interaction potential parameters.

Gas molecules	$\upvarepsilon \left(\frac{Kcal}{mol}\right)$	$\upsigma {\text{(\AA )}}$
N_2_-C_Graphenylene_	0.1049	3.495
CH_4_- C_Graphenylene_	0.1278	3.566
CO_2_- C_Graphenylene_	0.1639	3.495

In this section, we explore the movement of methane molecules adsorbed in pores with a size of 20 Å, at a temperature of 298 K and a methane pressure of 3.12 MPa. The injection gas pressure for both carbon dioxide and nitrogen in the left tank is 3.588 MPa. This pressure difference causes the injection gases to enter the pores, and subsequently, based on the interaction and adsorption capacity of the molecules with the pore walls, they displace methane molecules. The pressure calculations for the nanochannel and reservoirs were performed using the van der Waals equation of state. After determining the number of methane molecules present in the nanochannel, the calculated pressure was found to be 3.12 MPa. Consequently, the number of molecules placed in the reservoir was carefully selected to generate a slightly higher pressure compared to the nanochannel. Utilizing the Van der Waals equation of state, this quantity was calculated as 3.558, considering the given number of molecules and the volume of the reservoir.

**Figure 11:**
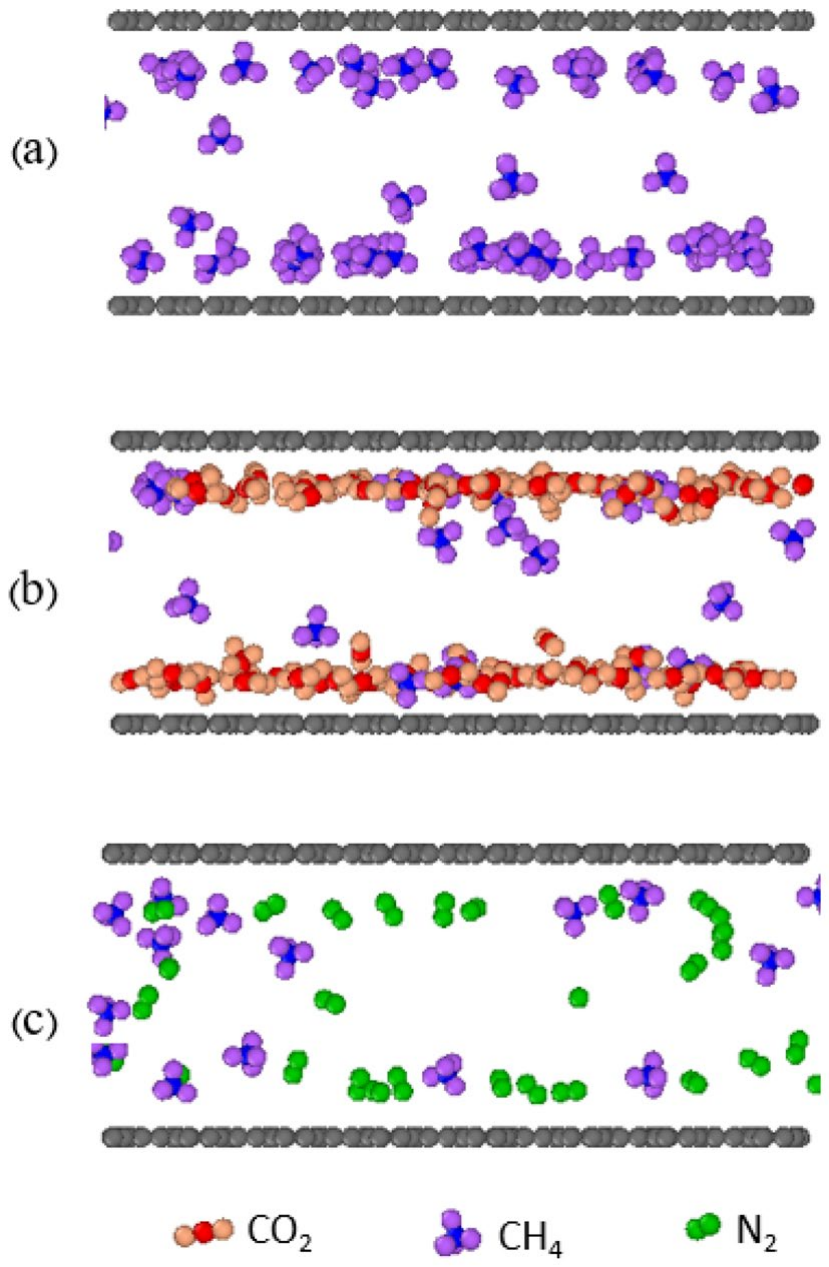
Adsorption images of molecules; (**a**) Primary adsorption of pure methane, (**b**) methane displaced by carbon dioxide, and (**c**) methane displaced by nitrogen.

Figure 11 illustrates the shape of the pore during the displacement process by the injection gases. Initially, only methane molecules are adsorbed in the pore under specific pressure and temperature conditions. Then, carbon dioxide and pure nitrogen are injected into the tank on the left. In this scenario, the injection gases enter the graphenylene pore, causing the initially adsorbed methane molecules to first move to the free phase in the center of the pore and then to be sent to the reservoir. When carbon dioxide gas is injected, carbon dioxide molecules first adsorb onto vacant sites. Then, some carbon dioxide molecules directly replace the adsorbed methane molecules because carbon dioxide has a stronger adsorption capacity than methane. Carbon dioxide molecules occupy low-energy positions, displacing methane molecules and storing them in the shale reservoir. Adsorbed methane molecules are released from the adsorption sites and return to the free phase. When nitrogen gas is injected, nitrogen molecules cannot directly replace the adsorbed methane molecules due to nitrogen's lower adsorption capacity compared to methane. However, nitrogen molecules can reduce the partial pressure of adsorbed methane molecules under constant pressure conditions. As the adsorption pressure decreases, the adsorption is also reduced, facilitating the displacement and desorption of methane molecules.

**Figure 12:**
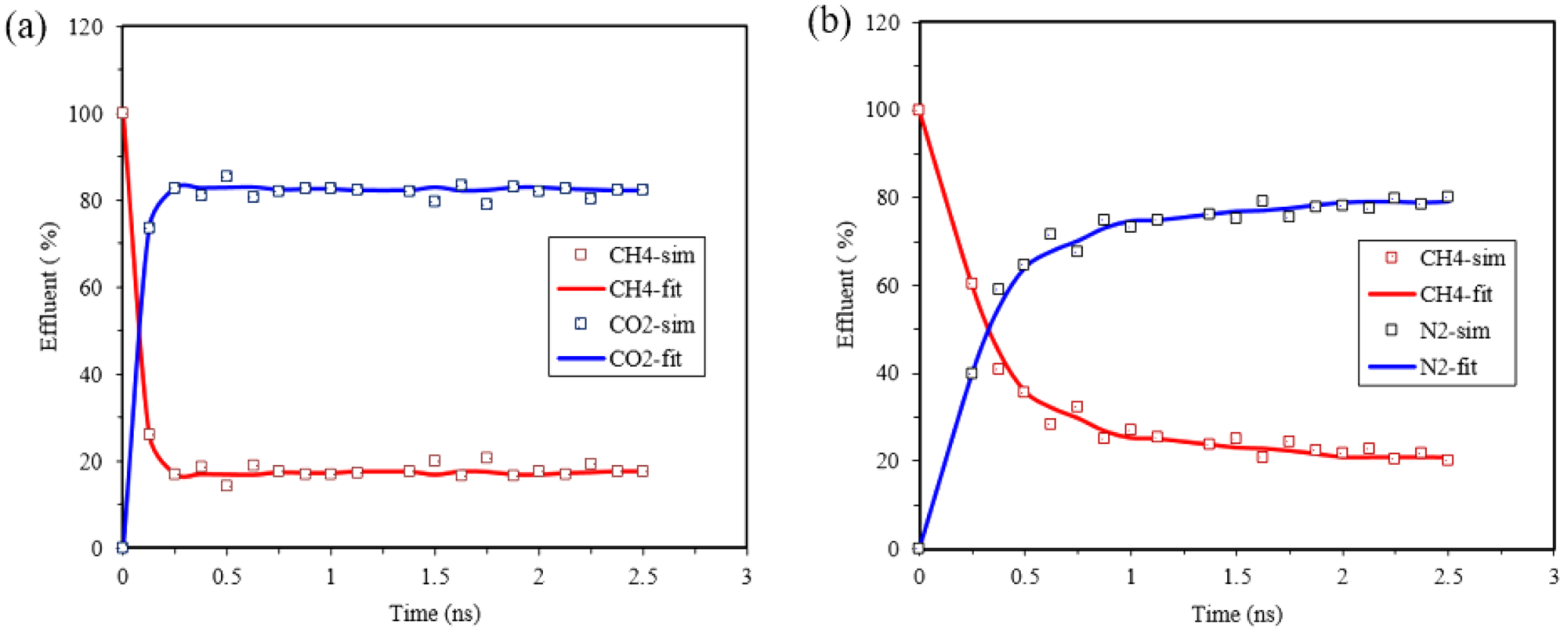
Displacement of carbon dioxide and pure nitrogen in methane; (**a**) pure carbon dioxide, (**b**) pure nitrogen.

Figure 12 depicts the percentage of gas concentration in graphenylene pores for methane, carbon dioxide, and nitrogen. Data points obtained from simulations are represented by solid lines fitting their trends. The desorption process is the reverse of surface adsorption. As shown in Fig. 12, the adsorbed methane molecules decrease rapidly when carbon dioxide and nitrogen gas are injected. Methane and carbon dioxide compete for adsorption on carbon surfaces, with carbon dioxide displaying a highly competitive adsorption ability due to its higher adsorption capacity compared to methane. When pure carbon dioxide is injected, the methane gas recovery and production curve in the graphenylene pore sharply declines, indicating displacement occurring within a short period. It eventually reaches a balance where the methane content reaches 17.24%.

In the case of pure nitrogen injection, the displacement and recovery curve of adsorbed methane molecules has a lower slope and a broader shape, indicating the lower capacity of nitrogen compared to methane. Nitrogen displaces methane by reducing the partial pressure, which takes longer for methane molecules to recover. Ultimately, the methane content in the pore reaches 20% Since carbon dioxide molecules are adsorbed in more pores and their quantity decreases in the free phase, their movement within the pores is slower. On the other hand, nitrogen, with its lower adsorption capacity compared to methane, is less strongly adsorbed in the pores, resulting in quicker movement. This increases the probability of molecular collisions, causing the adsorbed methane to move away from the surface. These findings align well with previous research^2,27,38^. The results demonstrate that both carbon dioxide and nitrogen can effectively displace adsorbed methane. These findings are expected to be highly valuable for the extraction and utilization of shale gas and carbon dioxide sequestration.

## Conclusion

Since methane is the primary component of shale gas and is adsorbed in shale pores, studying its adsorption behavior in shale reservoirs is crucial for evaluating shale gas resources. In this research, we investigated the mechanism of methane adsorption and displacement in cracks using molecular dynamics simulations. As the pore size increases, more methane molecules can enter the pore, while the methane density near the walls decreases. Once the adsorption sites on the pore surface become saturated, the molecules enter the bulk phase. With increasing pressure, the adsorption sites on the adsorbent surface fill up, reaching saturation at high pressure. Higher pressure also enhances the interaction between gas molecules and the wall surface, leading to increased adsorption. Gas adsorption in shale can be adequately described using the Langmuir adsorption isotherm model, despite the heterogeneity of gas adsorption sites in shale, considering physical and single-layer adsorption and increased adsorption with increasing pressure. Furthermore, it was observed that a higher number of methane molecules are adsorbed in smaller gap pores under relatively low pressure due to the smaller distance between the pore wall and the strengthened and adapted adsorption potentials, resulting in a stronger interaction.

As the temperature increases, the overall adsorption rate of methane molecules decreases due to increased gas molecule movement and greater resistance and collision between molecules.

The isosteric heat of methane adsorption increases with increasing pressure and decreases with increasing pore size due to enhanced molecular interaction. A higher isosteric heat indicates greater adsorption capacity and a stronger interaction between the adsorbed material and the adsorbent.

Carbon dioxide and nitrogen injection gases are utilized to displace adsorbed methane. Under the same conditions, the adsorption capacity of molecules follows the order CO2 > CH4 > N2, which indicates different displacement mechanisms that are compared.

During the displacement process, carbon dioxide molecules initially adsorb onto vacant sites and then directly replace adsorbed methane molecules due to their stronger adsorption capacity. This causes methane to enter the free phase. On the other hand, nitrogen can only be adsorbed in empty sites and displaces adsorbed methane by reducing the partial pressure.

The desorption curve for carbon dioxide exhibits a sharp shape and shorter transit time, while nitrogen shows a broader shape and longer transit time. It was determined that both carbon dioxide and nitrogen can effectively displace adsorbed methane.

## Data Availability

The datasets used and analyzed during the current study available from the corresponding author on reasonable request.
